# Baseline Elevations of Leukotriene Metabolites and Altered Plasmalogens Are Prognostic Biomarkers of Plaque Progression in Systemic Lupus Erythematosus

**DOI:** 10.3389/fcvm.2022.861724

**Published:** 2022-05-16

**Authors:** Sahar Baig, Kamala Vanarsa, Huihua Ding, Anto Sam Crosslee Louis Sam Titus, Maureen McMahon, Chandra Mohan

**Affiliations:** ^1^Department of Biomedical Engineering, University of Houston, Houston, TX, United States; ^2^Department of Medicine, David Geffen School of Medicine at the University of California, Los Angeles, Los Angeles, CA, United States

**Keywords:** atherosclerosis, biomarkers, blood, lupus, metabolites

## Abstract

Systemic lupus erythematosus (SLE) is associated with an increased incidence of acute and chronic cardiovascular disease as compared to the general population. This study uses a comprehensive metabolomic screen of baseline sera from lupus patients to identify metabolites that predict future carotid plaque progression, following 8–9 years of follow-up. Nine patients had SLE without plaque progression, 8 had SLE and went on to develop atherosclerotic plaques (SLE^PP^), and 8 patients were controls who did not have SLE. The arachidonic acid pathway metabolites, leukotriene B4 (LTB4) and 5-hydroxyeicosatetraenoic acid (5-HETE), and the oxidized lipids 9/13-hydroxyoctodecadienoic acid (HODE) were found to be significantly altered (*p* < 0.05 and fold-change >2) in SLE^PP^ patients compared to SLE patients without plaque progression. SLE^PP^ patients also exhibited significantly altered levels of branched chain amino acid (BCAA) metabolites and plasmalogens compared to the non-SLE controls. Taken together with the rich literature on these metabolites, these findings suggest that the identified metabolites may not only be prognostic of cardiovascular disease development in SLE patients, but they may also be active drivers of atheroma formation. Early identification of these high risk SLE patients may help institute preventive measures early in the disease course.

## Introduction

Systemic lupus erythematosus (SLE) is an autoimmune disease characterized by systemic inflammation due to pathogenic autoantibodies and pro-inflammatory immune signaling (1). SLE is chronic and may lead to significant deterioration in quality of life by way of many possible complications, the most ominous of which is end-stage organ failure, involving particularly the kidneys (2, 3). Many studies to date have focused on the renal manifestations of SLE, including lupus nephritis and end-stage renal disease, but there still remains much to be elucidated about the link between SLE and cardiovascular events (4). Cardiovascular disease (CVD) is the leading cause of death in the United States and Chung et. al postulate that up to 45% of SLE patients will have cardiovascular complications throughout their lifetimes, owing to systemic inflammation and heightened incidence of metabolic syndrome as compared to the general population (5–7). The incidence of emergent cardiovascular events may be up to twice as high in SLE patients when compared to those without SLE (8, 9). One longitudinal study of female patients found that those with SLE had a 4 fold increase in major adverse cardiovascular events when compared to healthy controls (10). Many patients are unaware that they have manifestations of CVD until they suffer an acute cardiovascular event, and these events are rapidly increasing in frequency such that they may overtake renal lupus as the most fatal complication associated with SLE (11). The profundity of the risk of cardiovascular disease in lupus patients is well-established in literature (8, 9, 12). Several studies have worked with cohorts of SLE patients to assess the longitudinal impact of lupus upon cardiac health, and have, in agreement with other studies, found that this population is much more at risk for developing cardiovascular complications than the general population. It is also well established that SLE may accelerate the formation of atherosclerotic plaques by promoting hypertension and hyperlipidemia (3, 13–15). While this relationship is clearly established in the literature, the mechanistic basis for this link at a molecular level is unclear. The objective of this study is to identify molecules whose baseline serum levels are significantly different in those SLE patients who will go on to develop atherosclerotic plaques vs. those who do not.

Several omics-based screens have been performed previously in order to explore the metabolic implications of systemic lupus erythematosus, including Wu et al., Perl et al., and Ouyang et al., but these have not examined cardiovascular disease (16–18). Omics-based screens have also been performed to identify biomarkers of cardiovascular disease in the general population, and suggest that a decrease in tricarboxylic acid cycle metabolites may be indicative of ischemic heart disease (19). Of particular interest, Leptin, pro-inflammatory high-density lipoprotein, and TNF related weak inducer of apoptosis (TWEAK) have been reported as potential biomarkers of atherosclerosis in SLE in previous studies (10, 20–22).

Information on the precise ways that SLE leads to cardiovascular disease may lead to the development of predictive tools to assess patients' risk of CVD early on when appreciable modifications in cardiovascular risk factors can still be instituted. In the present study, we have used a metabolomic screen to analyze baseline sera of patients with SLE to identify metabolites that are significantly different among patients who experience carotid plaque progression during follow-up compared to those who do not. Samples were analyzed using a metabolomic screen which used liquid chromatography-mass spectrometry (LC/MS) to detect levels of more than 3,000 metabolites. This analysis has illuminated the metabolic processes underlying pathogenesis of SLE complications, and has provided new insights on potential biomarkers for CVD in SLE. These findings may enable physicians to better identify SLE patients at risk for plaque progression, discuss and institute preventative measures and evaluate patients for long term therapy with agents such as statins and cholesterol lowering medications.

## Materials and Methods

### Patients and Samples

For the initial metabolomic screen, 25 serum samples were collected from patients at the University of California Los Angeles in Los Angeles, California. Of these samples, 8 were drawn from non-SLE controls, 8 from patients with SLE who went on to develop atherosclerotic plaque progression (referred to in this manuscript as “SLE^PP^”), and 9 from patients with SLE with no baseline carotid plaque or plaque progression during the follow-up period, with the mean follow-up duration being 8–9 years (Table 1). The controls were healthy except that some had hypertension or dyslipidemia (Table 1). None of the SLE patients in this cohort had active renal disease. The cohort had a mean age of 47–47.5 years and the SLE patients in this cohort, including those in the SLE^PP^ group, had a mean SLEDAI of 2. The patients in the SLE^PP^ and SLE control group were matched for ethnicity, history of GN, dyslipidemia, diabetes and tobacco use, as detailed in Table 1. All subjects included in this analysis are females. All studies were performed using samples obtained with informed consent with approval by the respective IRB boards at UCLA and UH.

**Table 1:** Demographics and clinical features of patient cohort used for metabolomic analysis.

	**SLE**	**SLE^**PP**^**	**Control**	***p*** **-value**
	**(*n* = 9)**	**(*n* = 8)**	**(*n* = 8)**	**SLE vs. SLE^**PP**^**
Age, years (median, range)	47.0 (36-60)	47.5 (31-56)	47.0 (22-63)	ns
Length of follow–up, months (median, range)	36.0 (26-45)	35.5 (25-44)	30.0 (23-43)	ns
Baseline SLEDAI	2.0 (0–8)	2.0 (0–26)	-	ns
Disease Duration (years)	8.0 (0–29)	9.0 (0–32)	-	ns
Race/Ethnicity				
Asian/Pacific Islander n (%)	3 (33.3%)	2 (25%)	2 (25%)	ns
African American n (%)	1 (11.1%)	2 (25%)	1 (12.5%)	ns
Caucasian n (%)	3 (33.3%)	2 (25%)	3 (37.5%)	ns
Hispanic n (%)	2 (22.2%)	2 (25%)	2 (25%)	ns
History of previous GN	3 (33.3%)	2 (25%)	-	ns
HTN	5 (55.6%)	0 (0)	3 (37.5%)	0.03
Dyslipidemia	3 (33.3%)	2 (25%)	2 (25.0 %)	ns
Diabetes	1 (11.1%)	1 (12.5%)	0	ns
History of previous tobacco use	5 (55.6%)	1 (12.5%)	2 (25%)	ns
History of baseline carotid plaque	0	1 (12.5%)	0	ns
Plaque progression over follow-up period	0	8 (100%)	0	ns

“*SLE^PP^” refers to SLE patients who exhibited plaque progression upon follow-up. The controls were healthy except that some had hypertension or dyslipidemia, as shown. All subjects were females. None had active renal disease*.

Carotid plaque status was assessed by ultrasound at baseline and during follow-up. As described in our previously studies (22), B-mode gray-scale, color, and spectral Doppler techniques were used to investigate the carotid arteries. All ultrasounds were performed by 4 registered vascular technologists, who were trained to perform the studies according to a preset protocol (23). The same radiologist interpreted all studies and was blinded with regard to the patients' demographic characteristics, SLE status, and any previous ultrasound results. The same ultrasound unit (Iu22; Philips Medical Systems) was used to scan all subjects.

The following anatomic sites were examined for the presence of atherosclerotic plaque, defined as the presence of focal protrusion into the arterial lumen with a thickness exceeding that of the surrounding wall of at least 50%: the bilateral common carotid, internal carotid, external carotid, and carotid bulbs. The number, location, and sonographic appearance of the plaques were recorded. This definition of plaque has been found to be an independent predictor of coronary heart disease events in the general population (24). None of the non-SLE controls had evidence of carotid plaques at baseline. All of the SLE^PP^ patients had no plaque at baseline except for one patient who had carotid plaque at baseline, and demonstrated evidence of plaque growth over the follow-up period (Table 1).

Metabolite difference effect sizes between SLE and controls in our previous studies have varied from 0.5 to 5.0 (16). Power calculations indicate that the selected group sizes are adequately powered (80%) to detect differences between the groups at the 0.05 level of significance for all metabolite differences with effect sizes 1.5 or larger.

### Metabolomic Screen

Samples were sent to Metabolon in Morrisville, North Carolina for a comprehensive metabolomic screen. Samples were prepared using the Hamilton Company's MicroLab STAR® system. Proteins in samples were precipitated by adding methanol and vigorously shaking for 2 min. After centrifugation of samples, proteins were extracted and dried using TurboVap®. Samples were then analyzed by LC/MS using Waters ACQUITY Ultra-Performance Liquid Chromatography (UPLC) equipped with heated electrospray ionization (HES-II) and an Orbitrap mass analyzer set at 35,000 mass resolution. Dried extracts were divided into fractions and reconstituted with different solvents depending on what mass spectroscopy method was to be used. Two fractions of proteins extracted from samples were analyzed by reverse phase UPLC with positive ion mode electrospray ionization (ESI). One fraction was analyzed by reverse phase UPLC with negative ion mode ESI. Finally, one fraction was analyzed by hydrophobic interaction liquid chromatography with negative ion mode. The ESI Scan range was 70–1,000 m/z and the library used included >3,000 metabolite standards. A total of 611 metabolites were identified in this study. In addition to the samples listed in Table 1, sera from three SLE subjects with renal SLE previously assayed using the same platform (16) were also included in this screen so that the present metabolomic findings can be bridged to previous metabolomic findings (16). Although these 3 subjects had no overt cardiovascular disease, they were not assessed for carotid plaques.

### Statistical Analysis

Following the metabolomic scan, Welch's two-sample *t-*test, a parametric test, was used to calculate *p-*values and to determine which metabolites were significantly altered (*p* < 0.05) among SLE patients with plaque progression (“SLE^PP^”) vs. the non-SLE controls or the SLE only group (“SLE”). Metabolomic scan data was also analyzed using Random Forest analysis, a machine learning algorithm, to determine which molecules were the best for discriminating between groups. As in a decision tree, the Random Forest algorithm (RFA) separates data based on chosen predictive variables and can be optimized to determine what characteristics of a given sample will lead to a specific outcome (25, 26). RFA builds upon predecessor predictive methods in that it incorporates random sampling of the data without replacement, at each node of each tree (26, 27). Many different random trees, collectively a random forest, can then be used to predict the outcome of a randomly sampled vector with relatively less susceptibility to over fitting than simple decision trees (26). By this method, we further determined which metabolites had the greatest predictive potential for plaque progression in patients with SLE.

To discern biological pathways that are significantly elevated in the sera of SLE^PP^ patients vs. control SLE patients who did not exhibit plaque progression, the metabolomic scan data was analyzed using “Reactome” from The Reactome Pathway Knowledgebase developed by Jassal et al. (https://reactome.org/userguide/analysis) (28). Receiver Operating Curves (ROC) were generated using the EasyROC software. Multiple cut-off thresholds are tested by the software in order to maximize the ROC area under curve “c” statistic. The corresponding sensitivity and specificity values are also documented.

## Results

### Significantly Dysregulated Metabolites in SLE Patients With Plaque Progression

**Figure 1:**
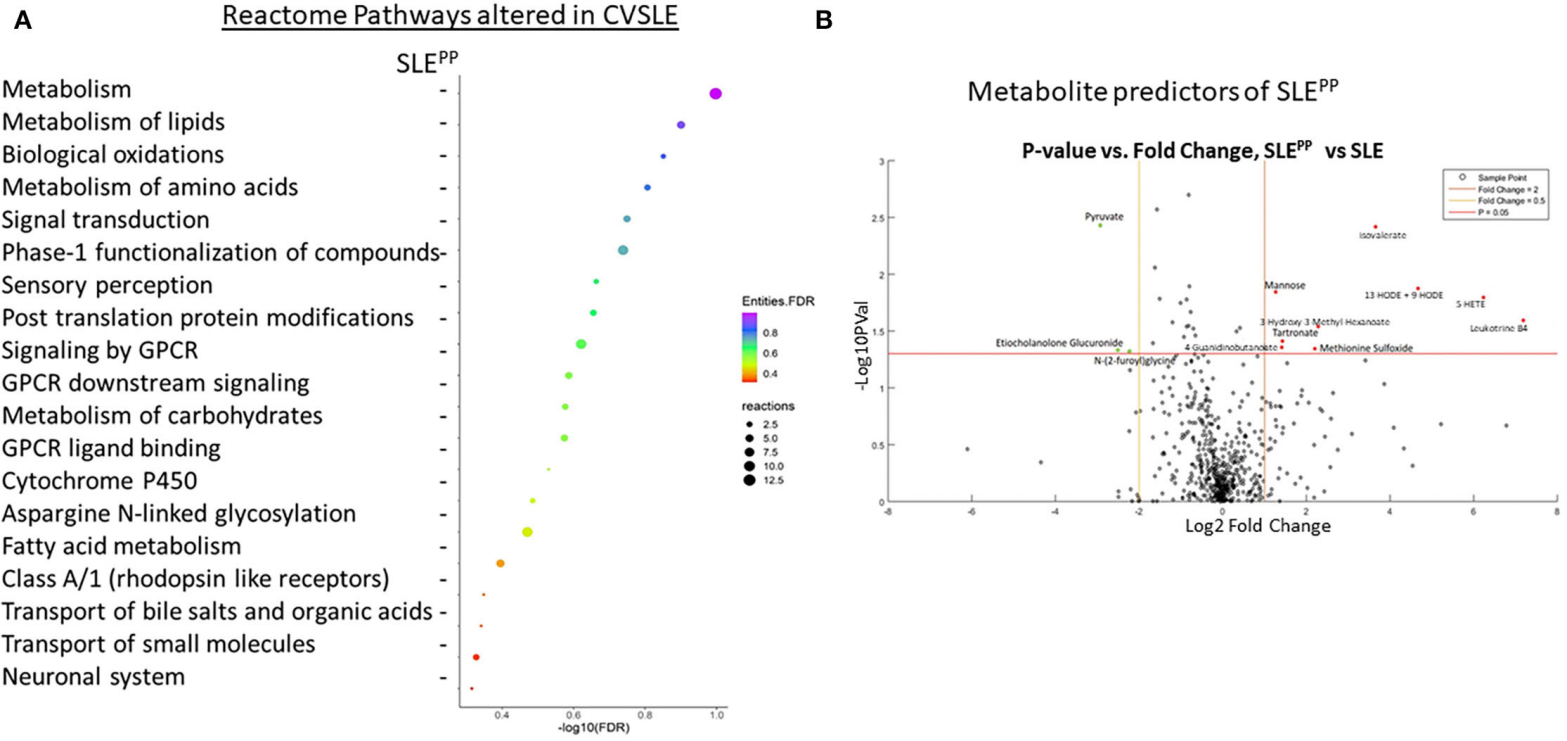
Functional pathways and molecules from serum metabolomics that best discriminate between SLE^PP^ and SLE patients include leukotriene metabolites, which are elevated in SLE^PP^, and plasmalogen metabolites, which are decreased in SLE^PP^. Baseline sera from SLE patients who did not develop atherosclerotic plaques, and SLE patients who later developed plaques (“SLE^PP^”) were subjected to comprehensive metabolomics, employing LC/MS. Shown in **(A)** are the metabolic pathways that included molecules significantly increased (*p* < 0.05) in the sera of SLE^PP^ vs. SLE patients, as determined by Reactome pathway analysis. For each pathway listed on the y-axis, the negative log base 10 of the false discovery rate is plotted on the x-axis. Dot colors correspond to the value of the inverse common log of the false discovery rate. Dot sizes correspond to “reactions”, or the degree of overlap between molecules of the pathway and molecules that were significantly elevated in SLE^PP^ sera as compared to SLE sera in the metabolomic screen. Pathways with the greatest proportion of molecules elevated in SLE^PP^ sera include metabolism of proteins, post-translational protein modification, and asparagine N-linked glycosylation. Pathways with the lowest false discovery rates included metabolism of lipids, biological oxidations, metabolism of amino acids and derivatives, signal transduction, and metabolism of proteins. Shown in **(B)** is a volcano plot of the most promising molecules from the metabolomic scan. Molecules represented in green were found to be decreased in concentration in SLE^PP^ vs SLE sera, molecules in red dots were elevated, and black molecules were not significantly altered. Among the serum metabolites found to be significantly elevated (*p* < 0.05 and fold change >2) in SLE^PP^ sera were leukotriene B4 (LTB4), 5-HETE, 13-HODE and 9-HODE, isovalerate, methionine sulfoxide, 3-hydroxy-3-methyl hexanoate, tartronate, 4-guanidinobutanoate, and mannose. Molecules that were decreased (*p* < 0.05 and fold change <0.5) in SLE^PP^ sera included pyruvate, etiocholanolone glucuronide, and N-(2-furoyl)glycine.

For the initial metabolomic scan, serum samples from 8 SLE patients who exhibited plaque progression (“SLE^PP^”) and 9 SLE patients who did not (“SLE”) were analyzed against 8 serum samples from controls. The metabolomic screen employed LC/MS based platforms and a library of over 3,000 metabolites. A total of 611 metabolites were identified in the study. Analysis of dysregulated metabolites in Reactome revealed which pathways were most significantly altered in SLE^PP^ vs. SLE, ordered by false discovery rates (Figure 1A). Of note were metabolism of lipids, biological oxidation pathways, metabolism of amino acids and proteins, and signal transduction. The pathways with the greatest “reactions”, or the greatest proportion of molecules within the pathway that overlapped with metabolites found in the metabolomic scan to be most significantly altered in SLE^PP^ vs. SLE, included metabolism of proteins, post-translational protein modification, and asparagine-N-linked glycosylation.

The most significantly dysregulated metabolites in baseline serum samples from SLE patients who went on to develop carotid plaques vs. those who did not are plotted as a volcano plot in Figure 1B. Of all molecules, the most significantly decreased among SLE^PP^ patients was pyruvate (*p* < 0.005 and fold change < 0.1). Etiocholanolone glucuronide and N-(2-furoyl)glycine were also decreased in SLE^PP^ sera with *p* < 0.05 and fold change <0.1. Among the most highly elevated molecules in SLE^PP^ sera was leukotriene B4 (LTB4) with a fold change >100, followed by 5-hydroxyeicosatetraenoic acid (5-HETE) with a fold change >64 and 9/13-hydroxyoctodecadienoic acid (9/13HODE) with a fold change >16. Isovalerate exhibited a fold change >8 and *p* <0.005. Other metabolites with a fold change >2 and *p* < 0.05 were 3-hydroxy-3-methyl-hexanoate, methionine sulfoxide, mannose, tartronate, and 4-guanidinobutanoate.

**Figure 2:**
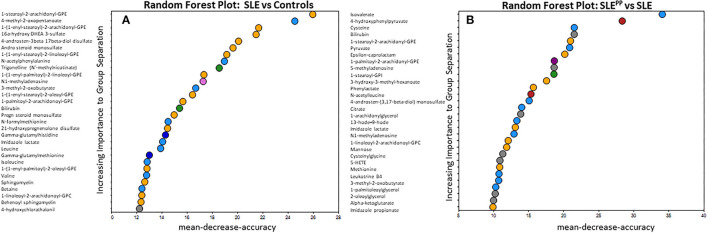
Random Forest analysis of molecules with highest potential to discriminate between SLE vs. healthy and SLE^PP^ vs. SLE shows that serum plasmalogens and branched chain amino acids have high importance to group separation. Baseline sera from SLE patients who did not develop atherosclerotic plaques, and SLE patients who later developed plaques (“SLE^PP^”) were subjected to comprehensive metabolomics. Identified metabolites were analyzed using a Random Forest algorithm, plotted in **(A,B)**. The molecules that exhibited the greatest utility in accurately discriminating between SLE vs. healthy sera were 1-stearoyl-2-arachidonoyl-GPE, 4-methyl-2-oxopentanoate, 1-(1-enyl-stearoyl)-2-arachidonoyl-GPE, 16 alpha hydroxy DHEA 3-sulfate, and 4-androstene-3beta, 17beta-diol sulfate. In **(B)**, molecules that exhibited predictive potential in distinguishing between SLE^PP^ and SLE are plotted. The five metabolites with the greatest importance to group separation and importance to accuracy of Random Forest predictions were isovalerate, 4-hydroxyphenylpyruvate, cysteine, bilirubin, and 1-stearoyl-2-arachidonoyl-GPE.

RFA of metabolites that best discriminated non-SLE controls from SLE patients revealed that glycerophosphoethanolamine (GPE) plasmalogens were significantly reduced among SLE patients as compared to controls (Figure 2A). These included 1-stearoyl-2-arachidonoyl-GPE, which additionally exhibited the greatest mean decrease in accuracy of all molecules plotted. 1-(1-enyl-stearoyl)-2-arachidonoyl-GPE was the GPE plasmalogen with the next highest importance to group separation and also exhibited the second highest mean decrease in accuracy. Other significantly reduced GPE plasmalogens were 1-(1-enyl-palmitoyl)-2-linoleoyl-GPE, 1-(1-enyl-stearoyl)-2-oleoyl-GPE, and 1-(1-enyl-palmitoyl)-2-oleoyl-GPE. Non-GPE plasmalogen molecules with high importance to group separation included 4-methyl-2-oxopentanoate, an alpha ketoacid; and 16-hydroxy dehydroepiandrosterone (DHEA) 3-sulfate, a sulfated steroid. Both were similarly reduced in SLE sera as compared to healthy sera.

In Figure 2B, metabolites were once again analyzed by RFA, but this time evaluated for their potential to discriminate between SLE^PP^ and SLE controls. Isovalerate, 4-hydroxyphenylpyruvate, cysteine and bilirubin were among those with the greatest importance to group separation. Several arachidonic acid derivatives were significantly elevated in SLE^PP^ sera when compared to SLE with no plaque progression. These include 1-palmitoyl-2-arachidonoyl-GPE, 13-HODE + 9-HODE, 1-linoleoyl-2-arachidonoyl-glycerophosphocholine (GPC), 5-HETE, and LTB4. The ketoacids 3-hydroxy-3-methyl-hexanoate, 3-methyl-2-oxobutyrate, and alpha-ketoglutarate were, by contrast, relatively lower in SLE^PP^ patients. Of the arachidonic acid metabolites most significantly altered in SLE^PP^ patients, 1-palmitoyl-2-arachidonoyl-GPE demonstrates the greatest predictive potential with a mean decrease in accuracy of 17, by RFA.

### Alterations in Metabolites of Arachidonic Acid

**Figure 3:**
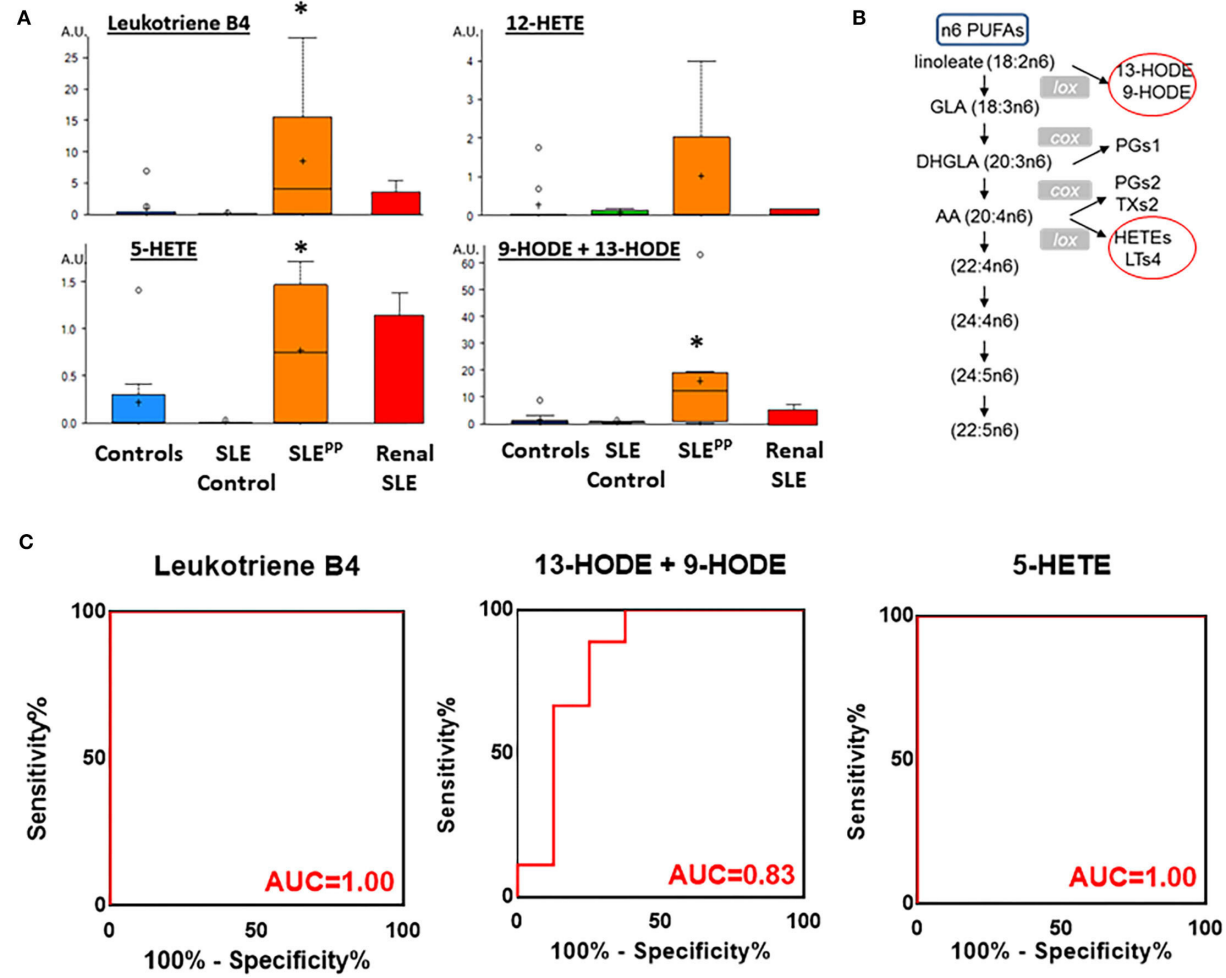
Pro-inflammatory lipoxygenase metabolites of n6-PUFAs (LTB4, 13-HODE+9-HODE, 5-HETE, and 12-HETE) were elevated in SLE^PP^ serum. Baseline sera from SLE patients who did not develop atherosclerotic plaques, and SLE patients who later developed plaques (SLE^PP^) were subjected to comprehensive metabolomics. Shown in **(A)** are bar charts (with dot plots shown in Supplementary Figure 1) of concentrations of various n6 polyunsaturated fatty acid (PUFAs) metabolites that were significantly elevated in SLE^PP^ compared to SLE and HC. Shown in red bars are the levels of the same metabolites in renal SLE in 10 subjects, as previously assayed (16). Metabolite comparisons between groups were carried out using a Welch's adjusted unequal variance *t*-test. * indicates *p* < 0.05. **(B)** illustrates the metabolic pathway of n6-PUFAs and arachidonic acid. Several of the metabolites in this pathway, including LTB4, 5-HETE, and 9/13-HODE, all of which are pro-inflammatory (circled red) were significantly elevated in SLE^PP^, relative to the other groups. LTB4 and 5-HETE had perfect accuracy in distinguishing SLE^PP^ from SLE **(C)**, both with 100% specificity and sensitivity values, while 9-HODE + 13-HODE exhibited 83% accuracy in distinguishing SLE^PP^ from SLE.

Many of the elevated molecules in SLE^PP^ patients shown in Figure 1B are part of the n6-polyunsaturated fatty acid (PUFA) and arachidonic acid lipoxygenase pathway. Thus, 9/13 HODE LTB4, 5-HETEand 12-hydroxyeicosatetraenoic acid (12-HETE) were elevated in SLE^PP^ patients when compared to SLE and controls (Figure 3A and Supplementary Figure 1). All four molecules are derived from n6 PUFA metabolism, as illustrated in Figure 3B. Among all SLE^PP^ patients interrogated in the metabolomic screen, the mean LTB4 concentration was 160.74 ng/mL, while the 9 SLE patients without plaque progression had a mean concentration of 16.55 ng/mL (*p* < 0.05), representing a nearly tenfold difference. 5-HETE and 9-HODE + 13-HODE similarly were found to be significantly (*p* < 0.05) elevated in SLE^PP^ patients when compared to SLE patients without plaque progression. 12-HETE was also distinctly elevated in SLE^PP^ sera. Interestingly, the elevations in these pro-inflammatory metabolites were higher in SLE^PP^ patients than in renal SLE (Figure 3A), with the latter being assayed in a previous metabolomic screen (16). Among these metabolites, LTB4 and 5-HETE had perfect accuracy in distinguishing SLE^PP^ from SLE, both with 100% specificity and sensitivity values (Figure 3C). In contrast, 9-HODE + 13-HODE exhibited 83% accuracy and 89% sensitivity in distinguishing SLE^PP^ from SLE.

### Alterations in Glycerophospholipids

**Figure 4:**
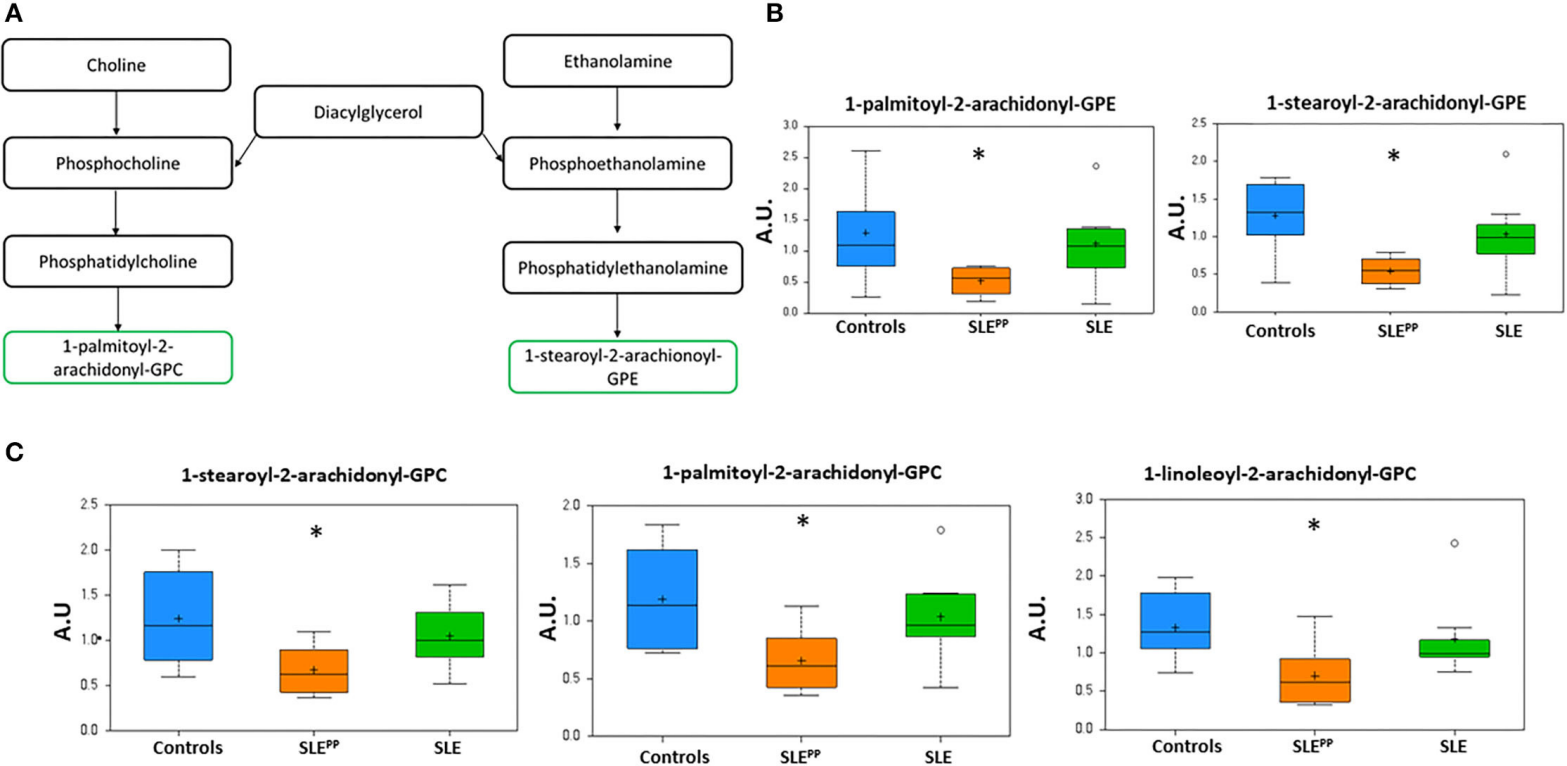
Phospholipids with 2-arachidonoyl acyl chains are reduced in SLE^PP^ serum. Phospholipid derivatives of diacylglycerol **(A)** were found to be reduced in SLE^PP^ serum samples as compared to SLE and non-SLE control samples. Some of the most discriminatory phospholipids are plotted in arbitrary units (AU), including the GPEs **(B)** and GPCs **(C)** highlighting the reduction in SLE, and the even more pronounced reduction in SLE^PP^. Indeed, these same metabolites were also highlighted as the most discriminatory metabolites for distinguishing SLE^PP^ from SLE by Random Forest analysis (Figure 2B). Baseline serum concentrations from 8 non-SLE controls are indicated in blue, 9 patients who exhibited plaque progression in orange, and 8 control SLE patients in green. Sera from SLE^PP^ patients contained significantly (*p* < 0.05) less 1-palmitoyl-2-arachidonoyl-GPE, 1-stearoyl-2-arachidonoyl-GPE, 1-stearoyl-2-arachidonoyl-GPC, 1-palmitoyl-2-arachidonoyl-GPC, and 1-linoleoyl-2-arachidonoyl-GPC than non-SLE controls and control SLE counterparts. Metabolite comparisons between groups were carried out using a Welch's adjusted unequal variance *t-*test. * indicates *p* < 0.05.

Figure 4A depicts the metabolic pathway of glycerophospholipids. Figures 4B,C demonstrate that 1-palmitoyl-2-arachidonoyl-GPE, 1-stearoyl-2-arachidonoyl-GPE, 1-stearoyl-2-arachidonoyl-GPC, 1-palmitoyl-2-arachidonoyl-GPC, and 1-linoleoyl-2-arachidonoyl-GPC were significantly decreased in SLE^PP^ sera when compared to both SLE and control sera. Indeed, these same metabolites were also highlighted as the most discriminatory metabolites for distinguishing SLE^PP^ from SLE by Random Forest analysis (Figure 2B).

### Alterations in Metabolites of Branched Chain Amino Acids

**Figure 5:**
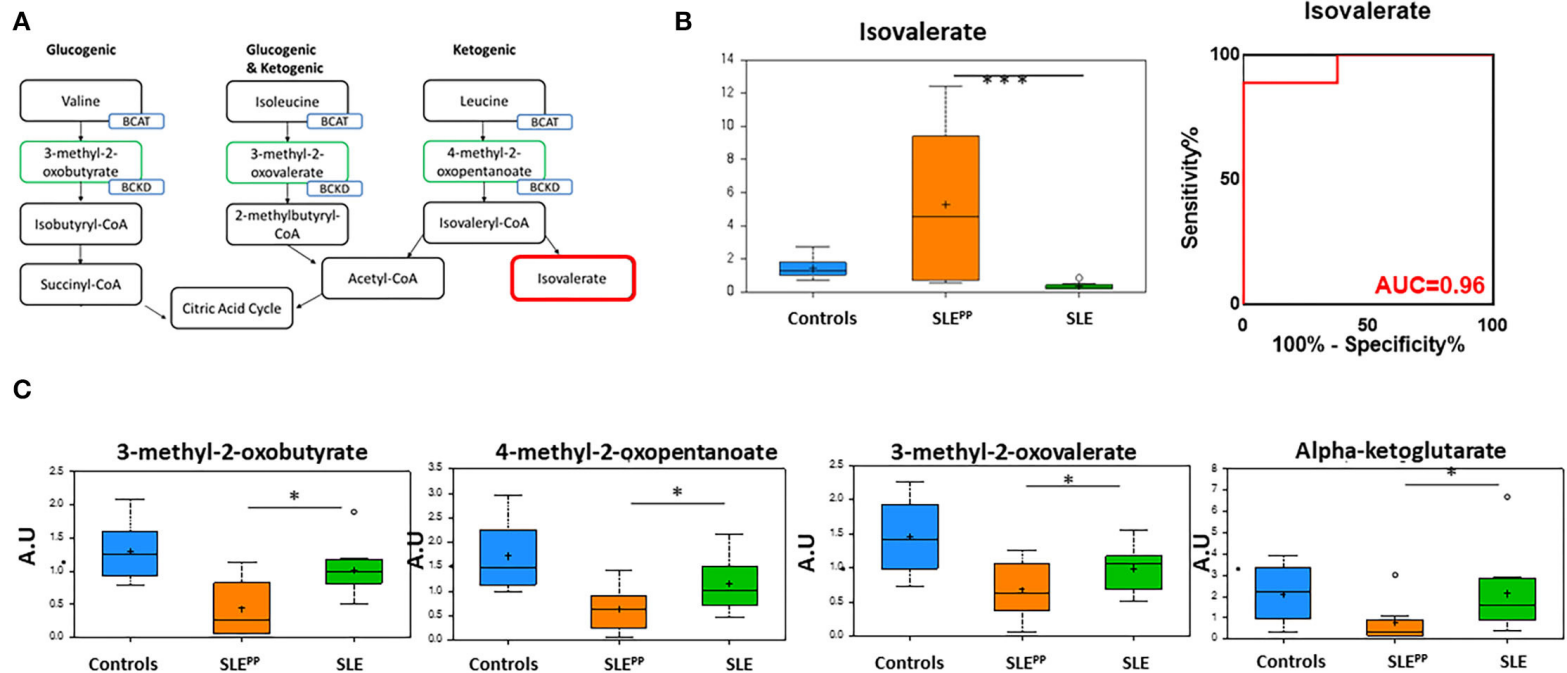
The alpha ketoacids isovalerate, 3-methyl-2-oxobutyrate, 4-methyl-2-oxopentanoate, 3-methyl-2-oxovalerate, and alpha ketoglutarate are altered in SLE^PP^ serum. Alpha ketoacid derivatives of branched chain amino acids (**A**; green boxes) were found to be reduced in SLE^PP^ serum when compared to SLE serum samples and non-SLE controls. Isovalerate (**A**; red box) was increased in SLE^PP^ sera. Serum levels of isovalerate are plotted in arbitrary units (AU) as a bar chart **(B)** where non-SLE controls are represented by the blue bar, SLE patients who went on to develop atherosclerotic plaques (SLE^PP^) by the orange bar, and SLE patients with no plaque progression by the green bar. The most significantly reduced alpha keto acids from the metabolic screen are plotted as bar charts in arbitrary units (AU) **(C)** where non-SLE controls are represented by blue bars, SLE^PP^ patients by orange bars, and SLE patients with no plaque progression by green bars. Valine, isoleucine, and leucine give rise to 3-methyl-2-oxobutyrate, 4-methyl-2-oxopentanoate, 3-methyl-2-oxovalerate, and alpha-ketoglutarate, all of which were significantly decreased in SLE^PP^ patients as compared to non-SLE controls and control SLE patients. Metabolite comparisons between groups were carried out using a Welch's adjusted unequal variance *t*-test. * indicates *p* < 0.05; *** indicates *p* < 0.001.

Figure 5A illustrates the metabolism of branched chain amino acids (BCAAs) which give rise to alpha keto acids. Random Forest analysis in Figure 2 also demonstrated that several alpha keto acid derivatives including alpha ketoglutarate were powerful discriminators between these groups and were decreased significantly in SLE^PP^ patients. Alpha ketoglutarate is a key player in the citric acid cycle and alpha keto acid derivatives of amino acids may be used as energy sources to be funneled into the citric acid cycle and substituted for alpha ketoglutarate (Figure 5A). Though one of the downstream products of BCAAs, isovalerate, was significantly elevated in SLE^PP^ (Figure 5B), the more proximal metabolites 3-methyl-2-oxovalerate, 3-methyl-2-oxobutyrate, and 4-methyl-2-oxopentanoate were uniformly decreased in SLE^PP^ sera (Figure 5C), alluding to the rapid consumption or turnover of these intermediate metabolites. Isovalerate exhibited 96% accuracy, 100% specificity and 89% sensitivity in distinguishing SLE^PP^ from SLE (Figure 5B).

### Alterations in Plasmalogen Metabolites

**Figure 6:**
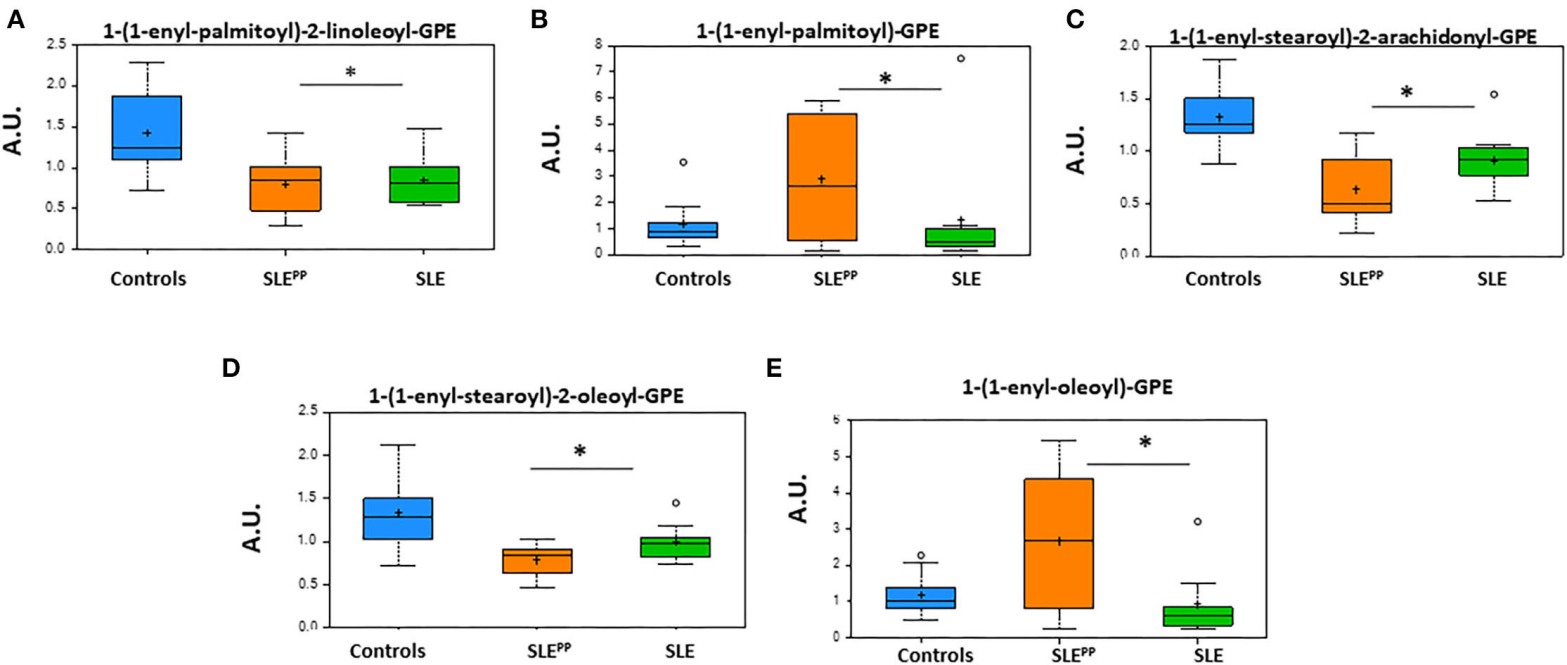
Glycerophosphoethanolamine derivatives of plasmalogens are altered in SLE serum. Indicated in the bar charts in arbitrary units (AU) are plasmalogen derivatives of fatty acids from the metabolic screen which were found to be altered in both SLE and SLE^PP^ sera when compared to non-SLE controls. Baseline serum concentrations from non-SLE controls are indicated in blue, SLE patients who exhibited plaque progression in orange, and control SLE patients in green. While 1-(1-enyl-palmitoyl)-2-linoleoyl-GPE, 1-(1-enyl-stearoyl)-2-oleoyl-GPE, and 1-(1-enyl-stearoyl)-2-arachidonoyl-GPE were reduced in the sera of both lupus groups as compared to the non-SLE controls, 1-(1-enyl-palmitoyl)-GPE and 1-(1-enyl-oleoyl)-GPE were highest in the SLE^PP^ group. All plotted molecules displayed the potential to discriminate between SLE^PP^ and SLE patients, with statistically significant (*p* < 0.05) differences in average serum concentrations. Metabolite comparisons between groups were carried out using a Welch's adjusted unequal variance *t-*test. * indicates *p* < 0.05.

Figure 6 summarizes alterations in key plasmalogen metabolites in SLE^PP^, SLE, and non-SLE control samples. While 1-(1-enyl-palmitoyl)-2-linoleoyl-GPE, 1-(1-enyl-stearoyl)-2-oleoyl-GPE, and 1-(1-enyl-stearoyl)-2-arachidonoyl-GPE were reduced in the sera of both lupus groups as compared to the non-SLE controls, 1-(1-enyl-palmitoyl)-GPE and 1-(1-enyl-oleoyl)-GPE were highest in the SLE^PP^ group. All plotted molecules displayed the potential to discriminate between SLE^PP^ and SLE patients, with statistically significant (*p* < 0.05) differences in average serum concentrations.

## Discussion

Literature has suggested for some time that lupus patients are more likely to suffer from cardiovascular complications than their healthy counterparts. This phenomenon was first studied longitudinally by Urowitz et. al., where it was found that atherosclerotic plaques and greater incidence of myocardial infarction were associated with later age of death in patients with active SLE (29). A study by Manzi et al. found that female SLE patients experienced earlier and more severe onset of cardiovascular disease than their healthy counterparts and were 50 times more likely to suffer a myocardial infarction (14). While many patients with SLE are now able to manage their symptoms in the short term, it is necessary to unravel mechanisms behind long-term implications of lupus such as renal and cardiovascular disease and begin searching for new approaches for early detection of such complications. To this end, it has been suggested that oxidized low-density lipoproteins, which have long been implicated in atherogenesis in the general population, may be used to gauge risk of atherosclerosis in patients with autoimmune disease (20). Our comprehensive metabolic screen aims to build upon the findings of such studies.

The present study represents the very first comprehensive OMICs study in SLE patients with plaque progression during longitudinal follow up. It is also the first attempt to comprehensively identify prognostic metabolites in SLE^PP^. Whereas some metabolites were significantly reduced in SLE patients, others were elevated, at baseline, before the emergence of atherosclerotic plaques. Most notable among the downregulated metabolites was the decrease in multiple alpha keto acids in SLE^PP^ sera, at baseline (Figure 4B). Alpha keto acid derivative byproducts of amino acid breakdown may originate from the liver or may be produced in the mitochondria during the tricarboxylic acid cycle. Both glucogenic and ketogenic amino acids can give rise to the derivatives which were decreased in SLE^PP^ patients (Figure 4A). This is in line with previous metabolomic findings which aimed to illuminate metabolic imbalances contributing to SLE in general (16).

In the present screen, alpha ketoglutarate and other alpha keto acids were found to be reduced in SLE patients compared to healthy subjects, pointing to potential upregulation of gluconeogenesis to feed an overactive Citric Acid Cycle in SLE (see Figure 5A), which may also explain the altered amino-acid and protein metabolism in SLE by Reactome analysis (Figure 1A), and the reported reductions in circulating amino acids in SLE (16). Increased consumption of alpha keto acids is also evidenced by the increased production of the downstream metabolite, isovalerate (Figures 5A,B). Isovalerate enhances chemotaxis and phagocytosis of granulocytes and monocytes (30, 31). Interestingly, isovalerate has previously been studied as a novel biomarker for diabetic nephropathy and endometrial cancer (32, 33). While the concentrations of alpha keto acids were found to be reduced in SLE serum, these molecules were further reduced in SLE^PP^.

Alpha-keto-acids promote differentiation of naïve CD4+ T cells to proinflammatory TH1 cells instead of immunosuppressive Treg cells (34). BCAA, which give way to alpha-keto-acids, are essential for lymphocyte function and altered BCAA metabolites cause impaired T cell activation (35, 36). The alpha ketoacids which were most significantly decreased are synthesized via reactions catalyzed by branched chain amino acid aminotransferase (BCAT) and are further catabolized via an irreversible attachment to coenzyme A which is catalyzed by branched chain alpha-keto acid dehydrogenase (BCKD). It may be postulated that the change in concentration of alpha keto acids in these groups may be due to an increase in BCKD or a decrease in BCAT activities, respectively. Indeed, evidence for such changes have been demonstrated in heart failure (37). Others have also postulated that a change in branched-chain amino acid catabolism is associated with cardiovascular disease, but it is unclear as to whether this is a cause or effect of the disease (38). Clearly, these postulates need to be experimentally verified in lupus as well.

In contrast, metabolites in the n6-PUFA/arachidonic acid lipoxygenase pathway were largely found to be elevated in SLE^PP^ sera in our metabolic screen. This is also in line with the literature, where the arachidonic acid pathway is a key player in the development of cardiovascular disease, and pro-inflammatory metabolites of n6-PUFA have been implicated in cardiovascular disease (39–41). Most notable is the increase in LTB4 concentration in SLE^PP^ sera, resonating well with previous reports in the cardiovascular disease literature (42, 43). Indeed, the role of LTB4 and related leukotrienes in inflammation and cardiovascular disease has been extensively documented (43–46). LTB4 causes activation of neutrophils, chemotaxis and adhesion of leukocytes to vascular endothelium and stimulates release of ROS and dsDNA from neutrophils (47–49). LTB4 also enhances the phagocytic activity of neutrophils and T-cell migration (50, 51). Together, these immune events may promote atheroma formation. In conjunction with our findings, this suggests that LTB4 may not only be prognostic of CVD in SLE, it is also likely to be an active driver of inflammation leading to atheroma formation (42, 47, 52, 53). Other metabolites in this pathway including 9-HODE and 13-HODE are also proinflammatory and have been implicated in atherogenesis (52–55). Wang et al. reported that the serum levels of 19 metabolites in conjunction with the use of traditional CVD risk factors was able to better predict which patients would go on to develop CVD than the use of risk factors alone (56). Their selected metabolites included 13-HODE and 9-HODE, which we also found significantly elevated in SLE patients who exhibited plaque progression.

Indeed, we had previously reported that LTB4, 5-HETE, 9-HODE and 13-HODE, all of which are metabolites in the same metabolic pathway (Figure 3B) are significantly elevated in SLE patients, associated with higher disease activity (16). 5-HETE is chemotactic for neutrophils and causes aggregation and degranulation of neutrophils (57–60). 12-HETE is involved in the recruitment and migration of neutrophils (61). High levels of 9/13 HODE have been shown to correlate with high SLE disease activity (16). 9/13-HODE are ligands of PPARg that enhances monocyte maturation and maintain macrophages in chronic inflammatory state (62). One can certainly envision this to promote atheroma formation. In the present study, we find that these same metabolites are highly upregulated only in SLE patients who later exhibited plaque progression, but not in patients who do not (Figure 3A). Taking together our previous metabolomic findings (16), the present metabolomic findings and the rich body of literature in CVD, it is clear that baseline levels of LTB4, 5-HETE, 9-HODE and 13-HODE are strong predictors and pathogenic drivers of future plaque progression and CVD. These findings also suggest that pharmacological inhibitors of this pathway, including LTB4 inhibitors, warrant evaluation in lupus patients bearing these serum biomarkers.

Plasmalogens emerge as another class of metabolites that may hold biomarker potential in SLE^PP^. In particular, the ethanolamine plasmalogens 1-(1-enyl-palmitoyl)-2-linoleoyl-GPE, 1-(1-enyl-stearoyl)-2-oleoyl-GPE, 1-stearoyl-2-arachidonoyl-GPE, and 1-(1-enyl-stearoyl)-2-arachidonoyl-GPE as well as the choline plasmalogen 1-palmitoyl-2-arachidonoyl-GPC were reduced in SLE^PP^ and SLE sera when compared to controls. Many of these molecules have been implicated in early colorectal and breast cancers and are being evaluated for their potential to detect these malignancies early (63, 64). Reduced plasmalogen levels impair phagocytosis by macrophages (65). Plasmalogen levels are reduced in SLE patients and are correlated with increased oxidative stress (66). Nguma et al., have suggested that ethanolamine plasmalogens may even serve a protective role against oxidative stress and have studied the utility of these molecules in the treatment of colitis in murine models (67). Levels of several choline plasmalogens were found by Nishimukai et al. to have a strong inverse correlation to progression of atherosclerosis and have even been suggested as possible biomarkers of CVD (68). Plasmalogens have also been proposed as potential biomarkers of SLE, as they have been found to be reduced in these patients (69). While our findings are consistent with the earlier reports, within our cohort these molecules are even further reduced in lupus patients who go on to develop atherosclerotic plaques. Further study will be necessary to assess the utility of plasmalogens as biomarkers for both SLE and SLE^PP^. Two plasmalogens, 1-(1-enyl-palmitoyl)-GPE and 1-(1-enyl-oleoyl)-GPE, were elevated in the SLE^PP^ group. In a 2018 study of patients with metabolic syndrome, Candi et al. found the same two ethanolamine plasmalogens to be elevated in the diseased population when compared to healthy controls (70). They had suggested that production of certain plasmalogens is elevated in response to the disease process, in order to combat inflammation (70, 71). Our findings in the present study are possibly explained by a similar mechanism, though experimental verification is warranted.

Several aspects of this study could be improved. As we have discussed previously, it would be important to control for the impact of medications, diet, genetics, and the gut microbiome in future evaluations of cardiovascular disease in SLE (71). In addition, the sample sizes of all groups studied need to be significantly increased. The patients used for this study tended to be older, and as such our findings may not be applicable to younger patients, though this needs to be experimentally verified. The clinical utility of these biomarkers as prognostic indicators may be limited to SLE patients with mild disease, as the mean SLEDAI score for both the SLE and SLE^PP^ groups was 2.0. Moreover, future studies must use larger cohorts to ascertain whether the reported results were confounded by variables such as tobacco use, which was most prevalent in the SLE group, or hypertension, which was present in the SLE and “HC” groups but not the SLE^PP^ group.

## Conclusion

Taken together with the large body of literature in the cardiovascular field that describe the same metabolites detailed here, the current observations offer resounding support for using baseline LTB4, 5-HETE, 9/13-HODE as well as altered alpha keto-acids and plasmalogens as prognostic biomarkers of long-term atherosclerosis and cardiovascular disease in patients with lupus, and for carefully evaluating the need for early institution of preventive measures in these high-risk subjects. Future studies are warranted to validate these results in larger patient cohorts.

## Data Availability Statement

The original contributions presented in the study are included in the article/Supplementary Material, further inquiries can be directed to the corresponding author/s.

## Ethics Statement

The studies involving human participants were reviewed and approved by IRB boards of UCLA, Los Angeles, CA and University of Houston, Houston, TX. The patients/participants provided their written informed consent to participate in this study.

## Author Contributions

MM and CM designed the study. MM provided patient samples. SB, KV, and HD performed the experiments. SB, KV, HD, AT, MM, and CM analyzed the data and wrote the manuscript. All authors have read the manuscript and concur with the conclusions.

## Funding

This work was supported by NIH R01 AR074096.

## Conflict of Interest

The authors declare that the research was conducted in the absence of any commercial or financial relationships that could be construed as a potential conflict of interest.

## Publisher's Note

All claims expressed in this article are solely those of the authors and do not necessarily represent those of their affiliated organizations, or those of the publisher, the editors and the reviewers. Any product that may be evaluated in this article, or claim that may be made by its manufacturer, is not guaranteed or endorsed by the publisher.

## References

[1] Choi J Kim ST Craft J. The pathogenesis of systemic lupus erythematosus—an update. Curr Opin Immunol. (2012) 24:651–7. 10.1016/j.coi.2012.10.004 23131610 PMC3508331

[2] Tan EM Cohen AS Fries JF Masi AT Mcshane DJ Rothfield NF. The 1982 revised criteria for the classification of systemic lupus erythematosus. Arthrit Rheumatism. (1982) 25:1271–7. 10.1002/art.1780251101 7138600

[3] Anders HJ Saxena R Zhao Mh Parodis I Salmon JE Mohan C. Lupus nephritis. Nat Rev Dis Primers. (2020) 6:7. 10.1038/s41572-019-0141-9 31974366

[4] Parodis I Gokaraju S Zickert A Vanarsa K Zhang T Habazi D. ALCAM and VCAM-1 as urine biomarkers of activity and long-term renal outcome in systemic lupus erythematosus. Rheumatology. (2020) 59:2237–49. 10.1093/rheumatology/kez528 31722419 PMC7449816

[5] Centers for Disease Control Prevention. (2013). Know the Facts About Heart Disease, 1–2. Available online at: https://www.cdc.gov/heartdisease/facts.htm (accessed December 24, 2021).

[6] Chung CP Avalos I Oeser A Gebretsadik T Shintani A Raggi P. High prevalence of the metabolic syndrome in patients with systemic lupus erythematosus: association with disease characteristics and cardiovascular risk factors. Ann Rheum Dis. (2007) 66:208–14. 10.1136/ard.2006.054973 16901956 PMC1798504

[7] Mok CC. Metabolic syndrome and systemic lupus erythematosus: the connection. Expert Rev Clin Immunol. (2019) 15:65–775. 10.1080/1744666X.2019.1620601 31094570

[8] Aviña-Zubieta JA To F Vostretsova K De Vera M Sayre EC Esdaile JM. Risk of myocardial infarction and stroke in newly diagnosed systemic lupus erythematosus: a general population-based study. Arthritis Care Res. (2017) 69:849–56. 10.1002/acr.23018 28129475

[9] Yurkovich M Vostretsova K Chen W Aviña-Zubieta JA. Overall and cause-specific mortality in patients with systemic lupus erythematosus: a meta-analysis of observational studies. Arthritis Care Res. (2014) 66:608–16. 10.1002/acr.22173 24106157

[10] Skaggs BJ Grossman J Sahakian L Perry L FitzGerald J Charles-Schoeman C. A panel of biomarkers associates with increased risk for cardiovascular events in women with systemic lupus erythematosus. ACR Open Rheumatol. (2021) 3:209–20. 10.1002/acr2.11223 33605563 PMC8063147

[11] Lupus Foundation of America. Common symptoms of lupus (2013). Available online at: https://www.lupus.org/resources/common-symptoms-of-lupus (accessed December 26, 2021).

[12] Svenungsson E Jensen-Urstad K Heimbrger M Silveira A Hamsten A de Faire U. Risk factors for cardiovascular disease in systemic lupus erythematosus. Circulation. (2001) 104:1887–93. 10.1161/hc4101.097518 11602489

[13] Sturfelt G Eskilsson J Nived O Truedsson L Valind S. Cardiovascular disease in systemic lupus erythematosus. A study of 75 patients form a defined population. Medicine. (1992) 71:216–23. 10.1097/00005792-199207000-00004 1518395

[14] Manzi S Meilahn EN Rairie JE Conte CG Medsger TA Jansen-McWilliams L. Age-specific incidence rates of myocardial infarction and angina in women with systemic lupus erythematosus: comparison with the Framingham Study. Am J Epidemiol. (1997) 145:408–15. 10.1093/oxfordjournals.aje.a009122 9048514

[15] Schiopu E Au KM McMahon MA Kaplan MJ Divekar A Singh RR. Prevalence of subclinical atherosclerosis is increased in systemic sclerosis and is associated with serum proteins: a cross-sectional, controlled study of carotid ultrasound. Rheumatology. (2014) 53:704–13. 10.1093/rheumatology/ket411 24357811 PMC4042927

[16] Wu T Xie C Han J Ye Y Weiel J Li Q. Metabolic disturbances associated with systemic lupus erythematosus. PLoS ONE. (2012) 7:e37210. 10.1371/journal.pone.0037210 22723834 PMC3378560

[17] Perl A Hanczko R Lai ZW Oaks Z Kelly R Borsuk R. Comprehensive metabolome analyses reveal N-acetylcysteine-responsive accumulation of kynurenine in systemic lupus erythematosus: implications for activation of the mechanistic target of rapamycin. Metabolomics. (2015) 11:1157–74. 10.1007/s11306-015-0772-0 26366134 PMC4559110

[18] Ouyang X Dai Y Wen J Wang L. 1H NMR-based metabolomic study of metabolic profiling for systemic lupus erythematosus. Lupus. (2011) 20:1411–20. 10.1177/0961203311418707 21976403

[19] Ussher JR Elmariah S Gerszten RE Dyck JR. The emerging role of metabolomics in the diagnosis and prognosis of cardiovascular disease. J Am Coll Cardiol. (2016) 68:2850–70. 10.1016/j.jacc.2016.09.972 28007146

[20] McMahon M Grossman J Chen W Hahn B. Inflammation and the pathogenesis of atherosclerosis in systemic lupus erythematosus. Lupus. (2006) 15:59–69. 10.1177/096120330607166816482750

[21] McMahon M Skaggs BJ Sahakian L Grossman J FitzGerald J Ragavendra N. High plasma leptin levels confer increased risk of atherosclerosis in women with systemic lupus erythematosus, and are associated with inflammatory oxidized lipids. Ann Rheum Dis. (2011) 70:1619–24. 10.1136/ard.2010.142737 21670088 PMC3147230

[22] McMahon M Skaggs BJ Grossman JM Sahakian L FitzGerald J Wong WK. A panel of biomarkers is associated with increased risk of the presence and progression of atherosclerosis in women with systemic lupus erythematosus. Arthritis Rheumatol. (2014) 66:130–9. 10.1002/art.38204 24449580 PMC4106468

[23] McMahon M Grossman J Skaggs B FitzGerald J Sahakian L Ragavendra N. Dysfunctional proinflammatory high-density lipoproteins confer increased risk of atherosclerosis in women with systemic lupus erythematosus. Arthritis Rheum. (2009) 60:2428–37. 10.1002/art.24677 19644959 PMC2753974

[24] Polak JF Szklo M Kronmal RA Burke GL Shea S Zavodni AE. The value of carotid artery plaque and intima-media thickness for incident cardiovascular disease: the multi-ethnic study of atherosclerosis. J Am Heart Assoc. (2013) 2:e000087. 10.1161/JAHA.113.000087 23568342 PMC3647272

[25] Schonlau M Zou RY. The random forest algorithm for statistical learning. Stata J. (2020) 20:3–29. 10.1177/1536867X20909688

[26] Breiman L. Random forests. Mach Learn. (2001) 45:5–32. 10.1023/A:1010933404324

[27] Hothorn T Lausen B. Double-bagging: combining classifiers by bootstrap aggregation. Patt Recogn. (2003) 36:1303–9. 10.1016/S0031-3203(02)00169-3

[28] Jassal B Matthews L Viteri G Gong C Lorente P Fabregat A. The reactome pathway knowledgebase. Nucleic Acids Res. (2020) 48:D498–503. 10.1093/nar/gkz1031 31691815 PMC7145712

[29] Urowitz MB Bookman AA Koehler BE Gordon DA Smythe HA Ogryzlo MA. The bimodal mortality pattern of systemic lupus erythematosus. Am J Med. (1976) 60:221–5. 10.1016/0002-9343(76)90431-9 1251849

[30] Wójcik R Małaczewska J Zwierzchowski G Miciński J Kaczorek-Łukowska E. The influence of dietary supplementation with the leucine metabolite β-hydroxy-β-methylbutyrate (HMB) on the chemotaxis, phagocytosis and respiratory burst of peripheral blood granulocytes and monocytes in calves. BMC Vet Res. (2020) 16:171. 10.1186/s12917-020-02389-1 32487098 PMC7268378

[31] Wójcik R Zabek K Małaczewska J Milewski S Kaczorek-Łukowska E. The Effects of β-Hydroxy-β-Methylbutyrate (HMB) on Chemotaxis, Phagocytosis, and Oxidative Burst of Peripheral Blood Granulocytes and Monocytes in Goats. Animals. (2019) 9:1031. 10.3390/ani9121031 31779122 PMC6940930

[32] Winther SA Mannerla MM Frimodt-Møller M Persson F Hansen TW Lehto M. Faecal biomarkers in type 1 diabetes with and without diabetic nephropathy. Sci Rep. (2021) 11:1–1. 10.1038/s41598-021-94747-8 34312454 PMC8313679

[33] Audet-Delage Y Villeneuve L Grégoire J Plante M Guillemette C. Identification of metabolomic biomarkers for endometrial cancer and its recurrence after surgery in postmenopausal women. Front. Endocrinol. (2018) 9:87. 10.3389/fendo.2018.00087 29593653 PMC5857535

[34] Klysz D Tai X Robert PA Craveiro M Cretenet G Oburoglu L. Glutamine-dependent α-ketoglutarate production regulates the balance between T helper 1 cell and regulatory T cell generation. Sci. Signal. (2015) 8:ra97. 10.1126/scisignal.aab2610 26420908

[35] Hayashi K Anzai N. L-type amino acid transporter 1 as a target for inflammatory disease and cancer immunotherapy. J Pharmacol Sci. (2022) 148:31–40. 10.1016/j.jphs.2021.09.006 34924127

[36] Calder PC. Branched-chain amino acids and immunity. J Nutr. (2006) 136:288S−93S. 10.1093/jn/136.1.288S 16365100

[37] Sun H Olson KC Gao C Prosdocimo DA Zhou M Wang Z. Catabolic defect of branched-chain amino acids promotes heart failure. Circulation. (2016) 133:2038–49. 10.1161/CIRCULATIONAHA.115.020226 27059949 PMC4879058

[38] Huang Y Zhou M Sun H Wang Y. Branched-chain amino acid metabolism in heart disease: an epiphenomenon or a real culprit? Cardiov Res. (2011) 90:220–3. 10.1093/cvr/cvr070 21502372 PMC3078803

[39] Kuehl FA Egan RW. Prostaglandins, arachidonic acid, and inflammation. Science. (1980) 210:978–84. 10.1126/science.6254151 6254151

[40] Sacks FM Campos H. Polyunsaturated fatty acids, inflammation, and cardiovascular disease: time to widen our view of the mechanisms. J Clin Endocrinol Metabol. (2006) 91:398–400. 10.1210/jc.2005-2459 16461954

[41] Simopoulos AP Leaf A Salem N. Workshop statement on the essentiality of and recommended dietary intakes for omega-6 and omega-3 fatty acids. Prostaglandins Leukot Essent Fatty Acids. (2000) 63:119–121. 10.1054/plef.2000.0176 10991764

[42] Helgadottir A Manolescu A Thorleifsson G Gretarsdottir S Jonsdottir H Thorsteinsdottir U. The gene encoding 5-lipoxygenase activating protein confers risk of myocardial infarction and stroke. Nat Genet. (2004) 36:233–9. 10.1038/ng1311 14770184

[43] Letts LG. Leukotrienes: role in cardiovascular physiology. Cardiovasc Clin. (1987) 18:101–13. 3038323

[44] Funk Colin D. Leukotriene modifiers as potential therapeutics for cardiovascular disease. Nat Rev Drug Discov. (2005) 4:664–72. 10.1038/nrd1796 16041318

[45] Fourie AM. Modulation of inflammatory disease by inhibitors of leukotriene A4 hydrolase. Curr Opin Investig Drugs. (2009) 10:1173–82. 19876785

[46] Thul S Labat C Temmar M Benetos A Bäck M. Low salivary resolvin D1 to leukotriene B4 ratio predicts carotid intima media thickness: a novel biomarker of non-resolving vascular inflammation. Eur J Prevent Cardiol. (2017) 24:903–6. 10.1177/2047487317694464 28195518

[47] Surmiak M Gielicz A Stojkov D Szatanek R Wawrzycka-Adamczyk K Yousefi S. LTB4 and 5-oxo-ETE from extracellular vesicles stimulate neutrophils in granulomatosis with polyangiitis. J Lipid Res. (2020) 61:1–9. 10.1194/jlr.M092072 31740445 PMC6939603

[48] Das UN. Current and emerging strategies for the treatment and management of systemic lupus erythematosus based on molecular signatures of acute and chronic inflammation. J Inflamm Res. (2010) 3:143–70. 10.2147/JIR.S9425 22096364 PMC3218729

[49] Mancuso P Nana-Sinkam P Peters-Golden M. Leukotriene B4 augments neutrophil phagocytosis of Klebsiella pneumoniae. Infect Immun. (2001) 69:2011–6. 10.1128/IAI.69.4.2011-2016.2001 11254552 PMC98124

[50] Costa MF de souza-Martins R de Souza MC Benjamin CF Piva B Diaz BL. Leukotriene B4 mediates gammadelta T lymphocyte migration in response to diverse stimuli. J Leukoc Biol. (2010) 87:323–32. 10.1189/jlb.0809563 19880577 PMC4057646

[51] Bäck M Bu DX Bränström R Sheikine Y Yan ZQ Hansson GK. Leukotriene B4 signaling through NF-kappaB-dependent BLT1 receptors on vascular smooth muscle cells in atherosclerosis and intimal hyperplasia. Proc Natl Acad Sci USA. (2005) 102:17501–6. 10.1073/pnas.0505845102 16293697 PMC1297663

[52] Alsalem M Wong A Millns P Arya PH Chan MS Bennett A. The contribution of the endogenous TRPV1 ligands 9-HODE and 13-HODE to nociceptive processing and their role in peripheral inflammatory pain mechanisms. Br J Pharmacol. (2013) 168:1961–74. 10.1111/bph.12092 23278358 PMC3623065

[53] Jira W Spiteller G. Dramatic increase of linoleic acid peroxidation products by aging, atherosclerosis, and rheumatoid arthritis. In: Honn KV, Marnett LJ, Nigam S, Dennis EA, editors. Eicosanoids and Other Bioactive Lipids in Cancer, Inflammation, and Radiation Injury, 4. Boston, MA: Springer (1999). p. 479–83. 10.1007/978-1-4615-4793-8_70 10667371

[54] Vangaveti V Baune BT Kennedy RL. Review: Hydroxy octadecadienoic acids: novel regulators of macrophage differentiation and atherogenesis. Ther Adv Endocrinol Metab. (2010) 1:51–60. 10.1177/2042018810375656 23148150 PMC3475286

[55] Warner DR Liu H Miller ME Ramsden CE Gao B Feldstein AE. Dietary linoleic acid and its oxidized metabolites exacerbate liver injury caused by ethanol via induction of hepatic proinflammatory response in mice. Am J Pathol. (2017) 187:2232–45. 10.1016/j.ajpath.2017.06.008 28923202 PMC5808136

[56] Wang Z Zhu C Nambi V Morrison AC Folsom AR Ballantyne CM. Metabolomic pattern predicts incident coronary heart disease. Arterioscler Thromb Vasc Biol. (2019) 39:1475–82. 10.1161/ATVBAHA.118.312236 31092011 PMC6839698

[57] Das UN. Lipoxins as biomarkers of lupus and other inflammatory conditions. Lipids Health Dis. (2011) 10:76. 10.1186/1476-511X-10-76 21569625 PMC3114772

[58] Aisen PS Haines KA Given W Abramson SB Pras M Serhan C. Circulating hydroxy fatty acids in familial Mediterranean fever. Proc Nat Acad Sci USA. (1985) 82:1232–6. 10.1073/pnas.82.4.1232 3919389 PMC397229

[59] Schwenk U Schröder JM. 5-Oxo-eicosanoids are potent eosinophil chemotactic factors. Functional characterization and structural requirements. J Biol Chem. (1995) 270:15029–36. 10.1074/jbc.270.25.15029 7797484

[60] O'Flaherty JT Thomas MJ Hammett MJ Carroll C McCall CE Wykle RL. 5-L-hydroxy-6,8,11,14-eicosatetraenoate potentiates the human neutrophil degranulating action of platelet-activating factor. Biochem Biophys Res Commun. (1983) 111:1–7. 10.1016/S0006-291X(83)80108-9 6403011

[61] Mrsny RJ Gewirtz AT Siccardi D Savidge T Hurley BP Madara JL. Identification of hepoxilin A3 in inflammatory events: a required role in neutrophil migration across intestinal epithelia. Proc Natl Acad Sci U S A. (2004) 101:7421–6. 10.1073/pnas.0400832101 15123795 PMC409934

[62] Nagy L Tontonoz P Alvarez JGA Chen H Evans RM. Oxidized LDL regulates macrophage gene expression through ligand activation of PPARγ. Cell. (1998) 93:229–40. 10.1016/S0092-8674(00)81574-3 9568715

[63] Liu T Tan Z Yu J Peng F Guo J Meng W. A conjunctive lipidomic approach reveals plasma ethanolamine plasmalogens and fatty acids as early diagnostic biomarkers for colorectal cancer patients. Expert Rev Proteomics. (2020) 17:233–42. 10.1080/14789450.2020.1757443 32306783

[64] Nguma E Yamashita S Han KH Otoki Y Yamamoto A Nakagawa K. Dietary ethanolamine plasmalogen alleviates DSS-induced colitis by enhancing colon mucosa integrity, antioxidative stress, and anti-inflammatory responses via increased ethanolamine plasmalogen molecular species: protective role of vinyl ether linkages. J Agric Food Chem. (2021) 69:13034–44. 10.1021/acs.jafc.1c04420 34723501

[65] Rubio JM Astudillo AM Casas J Balboa MA. Regulation of phagocytosis in macrophages by membrane ethanolamine plasmalogens. Front Immunol. (2018) 9:1723. 10.3389/fimmu.2018.01723 30087680 PMC6066501

[66] Hu C Zhou J Yang S Li H Wang C Fang X. Oxidative stress leads to reduction of plasmalogen serving as a novel biomarker for systemic lupus erythematosus. Free Rad Biol Med. (2016) 101:475–81. 10.1016/j.freeradbiomed.2016.11.006 27836780

[67] Tomida S Goodenowe DB Koyama T Ozaki E Kuriyama N Morita M. Plasmalogen deficiency and overactive fatty acid elongation biomarkers in serum of breast cancer patients pre- and post-surgery—new insights on diagnosis, risk assessment, and disease mechanisms. Cancers. (2021) 13:4170. 10.3390/cancers13164170 34439324 PMC8391794

[68] Nishimukai M Maeba R Ikuta A Asakawa N Kamiya K Yamada S. Serum choline plasmalogens—those with oleic acid in sn– 2—are biomarkers for coronary artery disease. Clin Chimica Acta. (2014) 437:147–54. 10.1016/j.cca.2014.07.024 25068205

[69] Candi E Tesauro M Cardillo C Lena AM Schinzari F Rodia G. Metabolic profiling of visceral adipose tissue from obese subjects with or without metabolic syndrome. Biochem J. (2018) 475:1019–35. 10.1042/BCJ20170604 29437994

[70] Scarfe GB Wright B Clayton E Taylor S Wilson ID Lindon JC. 19F-NMR and directly coupled HPLC-NMR-MS investigations into the metabolism of 2-bromo-4-trifluoromethylaniline in rat: a urinary excretion balance study without the use of radiolabeling. Xenobiotica. (1998) 28:373–88. 10.1080/004982598239489 9604301

[71] Zhang T Mohan C. Caution in studying and interpreting the lupus metabolome. Arthritis Res Ther. (2020) 22:172. 10.1186/s13075-020-02264-2 32680552 PMC7367412

