# Supplementary figures and images for: Baseline Elevations of Leukotriene Metabolites and Altered Plasmalogens Are Prognostic Biomarkers of Plaque Progression in Systemic Lupus Erythematosus
Source: Front Cardiovasc Med. 2022 May 16;9:861724. doi: 10.3389/fcvm.2022.861724 (PMC9149006; doi:10.3389/fcvm.2022.861724)

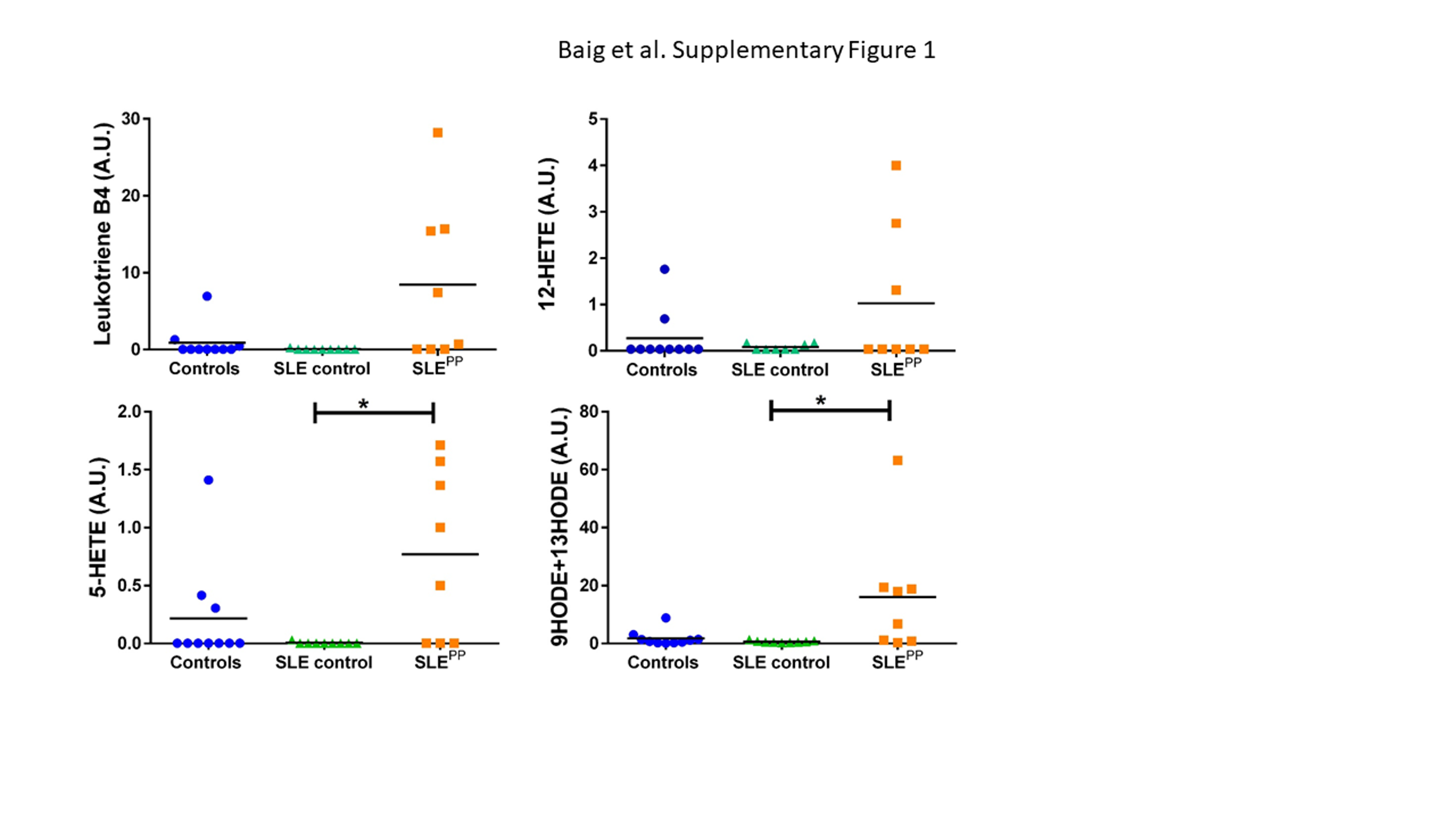

Supplement: Supplementary file 1 [file Image_1.TIF]
